# Subcritical Fluid Extraction of Chinese Quince Seed: Optimization and Product Characterization

**DOI:** 10.3390/molecules22040528

**Published:** 2017-03-25

**Authors:** Li Wang, Min Wu, Hua-Min Liu, Yu-Xiang Ma, Xue-De Wang, Guang-Yong Qin

**Affiliations:** 1 College of Physics Engineering, Zhengzhou University, Zhengzhou, Henan 450001, China; wanglihuina@163.com (L.W.); minwu2017@126.com) (M.W.); 2 Province Key Laboratory of Transformation and Utilization of Cereal Resource, Henan University of Technology, Zhengzhou, Henan 450001, China; 3 College of Food Science and Technology, Henan University of Technology, Zhengzhou, Henan 450001, China; myx366@163.com (Y.-X.W.); wangxuede1962@126.com (X.-D.W.)

**Keywords:** Chinese quince seed oil, subcritical fluid extraction, response surface methodology, characterization

## Abstract

Chinese quince seed (CQS) is an underutilized oil source and a potential source of unsaturated fatty acids and α-tocopherol-rich oil. Subcritical fluid (SCF) extraction is executed at lower pressures and temperatures than the pressures and temperatures used in supercritical fluid extraction. However, no studies on the SCF extraction of CQS oil are reported. Therefore, the objective of this study was to evaluate the use of SCF for the extraction of CQS oil and to compare the use of SCF with the classical Soxhlet (CS) and supercritical CO_2_ (SC-CO_2_) extraction methods. Response surface methodology (RSM) was used to investigate the extraction conditions: temperature (45–65 °C), time (30–50 min), and solvent/solid ratio (5–15 mL/g). The optimization results showed that the highest yield (27.78%) was obtained at 56.18 °C, 40.20 min, and 12.57 mL/g. The oil extracted by SCF had a higher unsaturated fatty acid content (86.37%–86.75%), higher α-tocopherol content (576.0–847.6 mg/kg), lower acid value (3.97 mg/g), and lower peroxide value (0.02 meq O_2_/kg) than extractions using CS and SC-CO_2_ methods. The SCF-defatted meal of oilseed exhibited the highest nitrogen solubility index (49.64%) and protein dispersibility index (50.80%), demonstrating that SCF extraction was a promising and efficient technique as an alternative to CS and SC-CO_2_ methods, as very mild operating conditions and an eco-friendly solvent can be used in the process with maximum preservation of the quality of the meal.

## 1. Introduction

A notable increase has recently been observed in the biochemical investigations of various oil seeds worldwide [1]. However, many of these inexpensive valuable wild plants have yet to be adequately utilized and investigated [2]. Many seeds of plants could be promising oil sources for nutritional, medicinal and industrial purposes. 

Chinese quince (*Chaenomeles sinensis*) belongs to the Rosaceae family, and the plants are distributed mainly in China, Japan, and Korea. Recently, many attempts have been made to verify the antiulcer and antioxidant activities of fruit extracts from the Chinese quince [3]. However, no reports in the literature of the oil content of Chinese quince seed (CQS) and its composition were found. Therefore, CQS has drawn increasing interest in recent years as a novel oilseed resource. 

For oil extraction, the methods generally used are expeller pressing and solvent extractions. However, the yield of the expeller pressing method is low. Solvent extraction involves long extraction times, and the subsequent separation at high temperature has a negative impact on oil quality. Although supercritical CO_2_ extraction reduced the use of organic solvents for oil extraction, the high cost of the process, the high pressure conditions and long extraction time could limit its application [4,5,6]. Therefore, a safer, more efficient, and cheaper method for extracting oil is in demand.

Subcritical fluid (SCF) extraction is one of the most popular techniques for extraction. According to the similar and compatible principle, the solvent and materials can effectively contact with each other, and in the molecule diffusion process, some components of the material are transferred to the solvent. After the extraction, the solvent can be removed by system depressurization at a low temperature and the small amount of residual solvent in the crude oils could be removed completely in the refining process [7]. The extraction is efficient, safe, and does not damage the heat-sensitive components of the materials [8]. Among various subcritical fluids used in SCF extraction, *n*-butane is used as the subcritical fluid mainly because it is carried out at lower temperatures and pressures for extraction compared with the SC-CO_2_ method. It also has excellent dissolving power for lipophilic compounds, and has a low boiling point, as well as being colorless, and cheap [9]. The advantages of SCF extraction can be summarized in three main aspects: (1) preservation of oil quality due to low extraction temperature; (2) preservation of the quality of the meal, with a high protein content; (3) reduction of the cost of utilities cost and total investment; and (4) low residual solvent is attributed to the reduction of the subsequent refining processes [7,9,10,11]. The subcritical *n*-butane extraction technique has also been used in the extraction of *Camellia* seed oil [8], *Nitraria tangutorum* seed oil [9], rice bran oil [12] and so on. To our knowledge, there has been no optimization study for extraction of CQS oil using SCF, which is important for scale-up purposes. 

Response surface methodology (RSM) was effectively utilized to assess the effects of multiple factors and their interactions on one or more variable quantities. RSM can reduce the number of experiments and provide a mathematical model [13]. For process optimization, the Box–Behnken design (BBD) has been widely used in the optimization of the SCF extraction parameters among the available experimental designs [7,9]. 

In the present study, the effects of extraction parameters (temperature, time, and solvent/solid ratio) on the CQS oil yield were investigated by RSM based on a BBD to obtain the highest oil yield. The widely accessible raw materials for the production of vegetable oils and the biodiversity of oilseed make the research attractive to the world, especially with regard to the optimization of the process, obtaining a product with unique quality and benefits. The physico–chemical characteristics and chemical compositions of CQS oil and protein meal products obtained using SCF extraction were evaluated, and the products obtained using hexane and SC-CO_2_ extractions were also comparatively investigated.

## 2. Results and Discussion

### 2.1. Oil extraction Process

**Figure 1:**
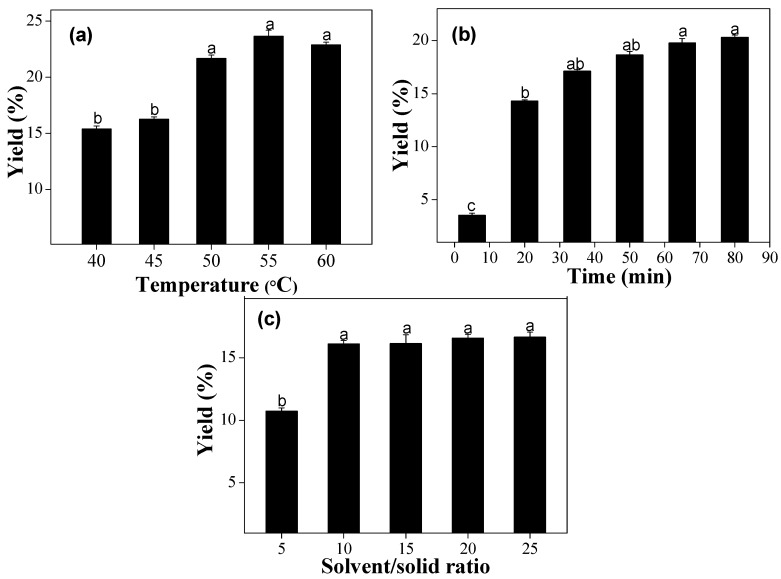
Effects of (**a**) extraction temperature, (**b**) extraction time, and (**c**) solvent/solid (S/S) ratio on oil yield from Chinese quince seed. Each value is the mean ± standard deviation (n = 3). Different letters of ^a–c^ in columns show statistically significant differences, *p* < 0.05.

A range of temperature treatments (40–60 °C) was employed for 30 min with a solvent/solid ratio (S/S ratio) of 10 mL/g to assess their effects on the extraction oil from CQS. Analysis of variance was used to verify the temperature experimental data, and the result was statistically significant at a 95% level of confidence. As shown in Figure 1a, the oil yield increased with increasing temperature, ultimately reaching a maximum at 55 °C. There was an increasing trend from 40 to 55 °C and this was probably due to the decrease in the solvent viscosity and the increase of the lipid solubility at higher temperatures. However, there was a decreasing trend from 55 to 60 °C, probably because the increase in temperature could lead to a large decrease in the subcritical *n*-butane density, with a consequent decrease in seed oil solubility [14]. Therefore, 55 °C was selected as the central point for temperature in the RSM experiments. 

Figure 1b shows the effect of extraction time on oil yield when other factors were set with an extraction temperature of 45 °C and an S/S ratio of 10 mL/g. The oil yield increased significantly in the initial 30 min, then slowed until reaching a constant value. Furthermore, the longer extraction time and long-term exposure to high temperatures could limit throughput on a large scale, while at the same time they potentially increased solvent loss due to vaporization [15]. Taking yield and processing costs into consideration, 30 min was sufficient for the extraction of seed oil, and thus the extraction time of 30 min was selected as the next experiment.

The effects of five S/S ratios on oil yield were studied, including 5, 10, 15, 20, and 25 mL/g at 45 °C for 30 min. Figure 1c shows that the yield of oil increased from 10.73% to 16.08% by raising the ratio from 5 to 10 mL/g. The result was in agreement with the principle of mass transfer, where the driving force was the concentration gradient between the solid and liquid during the mass transfer, which increased at a higher S/S ratio [16]. However, an excessive increase of the S/S ratio did not significantly increase oil yield. A larger amount of solvent would not change the driving force. Considering economics, an S/S ratio of 10 was appropriate for the central point in the RSM experiments.

As to response surface optimization of SCF extraction conditions, the data from 17 experiments are represented in Table 1. The yield of CQS oil was fitted to multiple regressions. The analysis of variance (ANOVA) results are shown in Table 2. The low *p* value of the model (< 0.0001) revealed that the model was statistically significant. The analysis of variance also showed that there was a non-significant lack of fit, which further validates the model (*p* > 0.05). The polynomial model fitted to the experimental data has been highly significant (*p* < 0.05). The coefficient of determination (*R*^2^) was 0.9972; thus, the model explained most of the observed variation. The adjusted *R*^2^ (0.9937) was in good agreement with the *R^2^*, suggesting an excellent correlation between the predicted values and the experimental results. The coefficient of variation (CV) of 1.87% indicated that the model was reproducible. A second-order polynomial function was built to describe the effects of the independent factors, namely, temperature (*X_1_*), time (*X_2_*), and S/S (*X_3_*), on the dependent variable oil yield (*Y*), which was given as follows:

*Y* = 24.78 + 6.75*X_1_* + 0.76*X_2_* + 3.53*X_3_* – 1.47*X_1_X_2_* + 0.78*X_1_X_3_* + 0.47*X_2_X_3_* – 5.76*X_1_^2^* – 2.30*X_2_^2^* – 3.92*X_3_^2^* (1)

**Figure 2:**
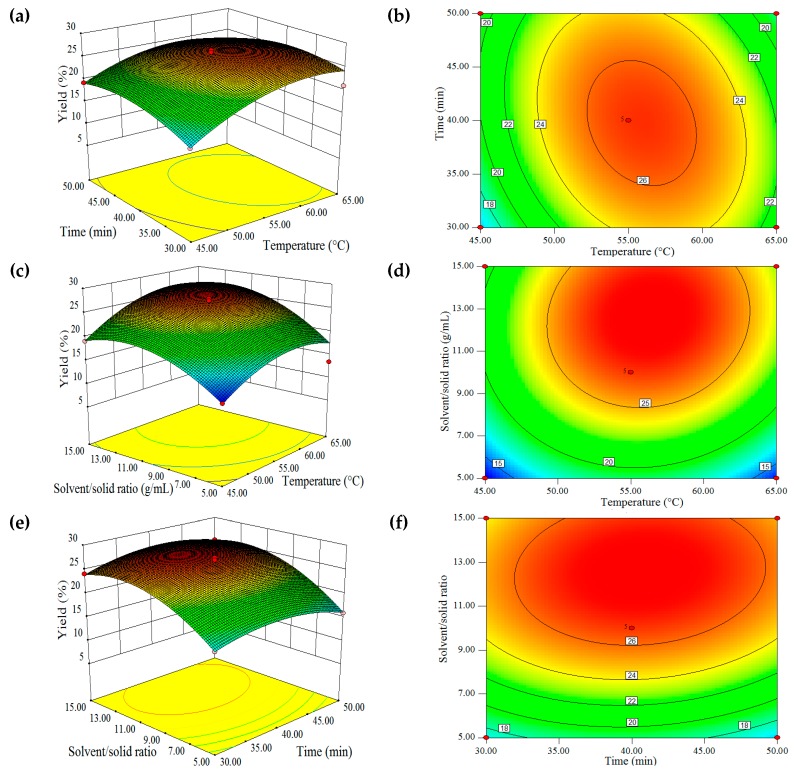
Response surface plots of oil yield affected by extraction temperature, extraction time and solvent/solid ratio. (**a**,**b**): time and temperature; (**c**,**d**): solvent/solid ratio and temperature; (**e**,**f**): solvent/solid ratio and time.

Based on the information (Equation (1)) obtained by using RSM, the 2D contours and 3D response surface plots were generated to measure the levels of the processing variables to achieve the optimal yield of CQS oil (Figure 2). With the S/S ratio set to 10 mL/g, the highest oil yield was obtained by an extraction time of 33.8–45.0 min and the extraction temperature range of 52.3–59.4 °C (Figure 2a,b). By setting the extraction time to 40 min, the optimal oil yield should be in the S/S ratio range of 8.3–15.0 mL/g with extraction temperature between 44.3 and 58.0 °C (Figure 2c,d). With the extraction temperature set at 55 °C, the oil yield should reach its optimal value between the S/S ratio of 9.1–15.0 mL/g and the extraction time of 32.0–49.0 min (Figure 2e,f). 

By employing the Design-Expert software, the optimal values of the three variables were calculated to be 56.18 °C, 12.57 mL/g, and 40.20 min for extraction temperature, S/S ratio, and the extraction time, respectively. The maximum predicted CQS oil yield was 27.78% under the optimal conditions. For operational convenience, the optimal conditions were 56 °C, 13 mL/g, and 40 min. Three verification experiments were carried out under the optimum conditions to confirm the adequacy of the predicted model. A mean value of 27.68 ± 0.12% was obtained from laboratory experiments. There were no significant differences (*p* > 0.05) between the predicted and experimental values. Therefore, the results indicated the success of RSM in the optimization of CQS oil yield by SCF extraction.

### 2.2. Oil Characterization

#### 2.2.1. Total and 2-Position Fatty Acid Composition

Table 3 showed that the main fatty acid components of these seed oils were linoleic acid and oleic acid, and the total concentrations of linoleic acid and oleic acid in the oils extracted by three methods were 85.58% (classical Soxhlet, CS), 85.99% (SCF), and 84.81% (supercritical CO_2_, SC-CO_2_), respectively, higher than the sum of linoleic and oleic acids detected in peanut oil samples [17]. All the extraction oils by three methods were rich in unsaturated fatty acids, which played an important role in the regulation of a variety of physiological and biological functions in the living organisms and were relatively low in saturated fatty acids. Chinese quince seeds have high unsaturated fatty acids such as linoleic and oleic acids, which have cardioprotective effects and are able to reduce the level of cholesterol in the blood [18]. The unsaturated fatty acids represented 86.37%–86.75% of the total fatty acids present in CQS oil, higher compared with argan oil (81%–81.7%) [19], peanut oil [17], and these made quince seeds a compelling choice for the functional foods market. There were no important impacts on fatty acid composition among the seed oils obtained by three methods, which indicated that the content was virtually the same regardless of the extraction method used. Unsaturated fatty acids occupied almost 98.41% (CS), 90.97% (SCF), and 97.01% (SC-CO_2_) of the Sn-2 position of the glycerol backbone. Statistically significant differences were observed in the content of arachidic acid. The results demonstrated that unsaturated fatty acids of CQS oil were preferentially located at the Sn-2 position. 

#### 2.2.2. Physico-Chemical Determinations

The physico-chemical characteristics of the extracted CQS oils were affected by the extraction methods (Table 4). As a result, a significant difference in colour could be found. The lovibond tintometer colour value of oil extracted by SCF was 95.5 Lovibond colour units, surprisingly high compared with oil extracted by SC-CO_2_ (82.0 Lovibond colour units). The relatively low polarity of *n*-butane, compared with the polarity of SC-CO_2_, assisted in the rapid solubilization of both oil- and fat-soluble pigment. However, oil extracted by CS had the highest colour index (99.4 Lovibond color units) with an increase in the red and yellow colour components. The low solubility of CQS pigment in SC-CO_2_ suggested that oil extracted in this way would show less tendency undergo colour fixation than CS-extracted oil. 

The densities and refractive indices of oils extracted by CS, SCF, and SC-CO_2_, respectively, showed no significant differences (*p* > 0.05).The peroxide and acid values were very important for various extracted oils because they showed the information about the application and composition of CQS oil. As a result (Table 4), a significant difference in acid values could be found. Acid values were 3.97 ± 0. 04 (SCF), 4.75 ± 0.05 (SC-CO_2_) and 6.87± 0.12 (CS) mg/g, respectively. The acid value of SC-CO_2_-extracted oil was lower compared with CS-extracted oil. This result was similar to the report about Mexican chia seed oil extracted by SC-CO_2_ [20]. SCF-extracted oil still possessed a very low acid value without extra protection, which indicated its good quality for potential food applications. The results also revealed that the three extraction methods provided very stable CQS oils with the reliable stability (peroxide value < 0.1 meq O_2_/kg). SCF-extracted oil showed the lowest peroxide value (0.02 meq O_2_/kg), and this value could be attributed to the lowest extraction temperature among three methods. The results indicated that the SCF extraction process could be a useful method for producing high-quality CQS oil. One of the major concerns for industrial applications was the formation of peroxides in the oils, and the low peroxide value of CQS oil met the necessary requirements. Iodine values (IVs) of oils obtained by CS (107.61 ± 1.19 g/100 g) and SCF (108.91 ± 1.20 g/100 g) methods were slightly lower than IVs of oils obtained by the SC-CO_2_ (110.27 ± 5.13 g/100 g) method. The IVs were similar due to the similar fatty acid compositions among the three oils [21]. The saponification values of CQS oils (190.72 ± 0.79, 191.25 ± 0.88 and 193.76 ± 0.91 mg KOH/g obtained by CS, SCF, SC-CO_2_ methods, respectively) were similar to the saponification values of olive oil (191.93 mg KOH/g) [22], indicating a high content of low molecular weight triacylglycerols. Unsaponifiable matter of SC-CO_2_-extracted oil (1.61 ± 0.002 g/100 g) showed a higher value compared with CS-extracted oil (0.91 ± 0.001 g/100 g) and SCF-extracted oil (1.35 ± 0.006 g/100 g), which indicated an efficient extraction of unsaponifiables using SC-CO_2_. 

The induction time for the oil extracted by SCF (6.85 ± 0.07 h) was longer than the induction times of the oils extracted by SC-CO_2_ (0.33 ± 0.00 h) and CS (6.77 ± 0.04 h) methods. The longer induction time suggested stronger antioxidant activity. The oxidative stability of SCF-extracted oil was higher than the oxidative stabilities of SC-CO_2_ and CS-extracted oils. The longer induction time for SCF-extracted oil was due to higher level of total tocopherols. The result proved that long times of extraction using the CS method under heating favoured the peroxidation reaction, and hydrolysis resulted in a shorter induction time [23].

#### 2.2.3. Tocopherol Composition

Among all Vitamin Es, α-tocopherol presents the highest biological potency [24]. Total tocopherol content of SCF-extracted oil was 851.3 ± 0.23 mg/kg, which was higher than the total tocopherol content of CS-extracted oil (620.1 ± 0.21 mg/kg) and the total tocopherol content of SC-CO_2_-extracted oil (578.4 ± 0.28 mg/kg). The results revealed high amounts of tocopherols in the CQS oil (578.4–851.3 mg/kg) which was a better tocopherol source compared with common oils such as olive (17.0 mg/100 g), soybean (49.7 mg/100 g) [25] and so on. According to our results and other reports, the α-tocopherol content (576.0–847.6 mg/kg) of CQS oil was higher than the other woody plant oils such as olive (1–240 mg/kg), palm (180–260 mg/kg), walnut (560 mg/kg) and tea seed (210 mg/kg) [26].

These results showed that the mass transfer of tocopherol is closely connected to its solubility in the oil that contained its initial amount present in the matrix and the subsequent consumption of the solid phase [27]. Tocopherols are nonpolar molecules due to their long side chain and aromatic rings; therefore, they are quite soluble in *n*-hexane and subcritical *n*-butane solvents. The relatively low polarity of *n*-butane, compared to the polarity of SC-CO_2_, assisted in the rapid solubilization of both oil and fat-soluble α-tocopherol. The results indicated that under mild conditions subcritical *n*-butane was superior to SC-CO_2_ for the extraction of tocopherols [28]. 

#### 2.2.4. Thermal Stability

**Figure A1:**
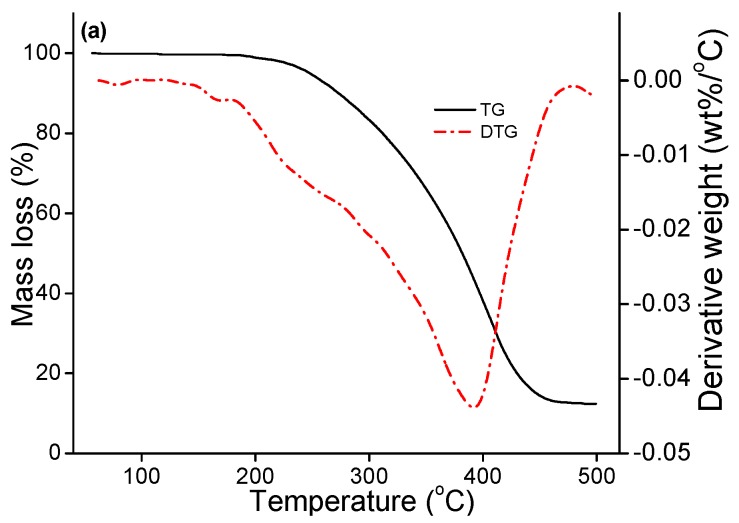

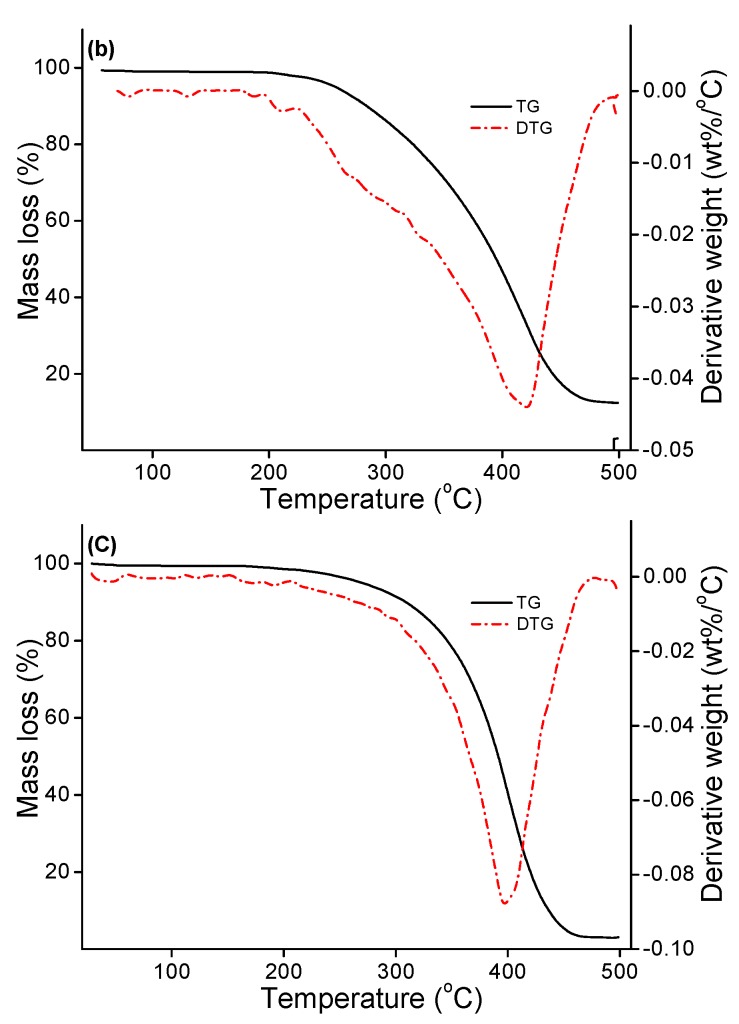
Thermogravimetric/derivative thermogravimetric (TG/DTG) curves of Chinese quince seed oils extracted using (**a**) classical Soxhlet, (**b**) subcritical fluid, (**c**) supercritical carbon dioxide.

The thermogravimetric (TG) and derivative thermogravimetric (DTG) curves for the analysis of the oils obtained by three different extraction methods are shown in Figure A1. From these curves, distinct reaction regions were identified for the oxidation of the crude CQS oils. The first region for the oil extracted by CS identified in the DTG curve showed a first peak at approximately 170 °C (Figure A1a), which occurred typically due to volatility and moisture losses [29]. Then, a continuous, smooth and steep descent line revealing a rapid loss of mass was found in the TG curve. The highest peak of weight loss observed in the DTG curve (at approximately 390°C) may be contributed to by distillation, oxidation reactions and bond scission. Oxygen molecules could bind with hydrocarbon molecules to produce various oxygenated substances such as ketones, aldehydes, and acids that could offset distillation and evaporation effects, leading to the reduction of oil mass and loss diversity in air [30].

The same approach was used to study TG and DTG results for the other oils obtained by SCF and SC-CO_2_ extraction methods (Figure A1b,c). The first regions of SCF and SC-CO_2_ extractions showed, respectively, first peaks at approximately 200 °C and 190 °C, and the highest peaks of weight loss were observed at approximately 420 °C and 400 °C, respectively.

DTG plots in a differential form for CS, SCF, and SC-CO_2_ were prepared to discuss the possible weight/mass loss of the sample corresponding to the temperature. Onset temperature was defined as the temperature at which sample decomposition began. SCF-extracted oil had a higher initial decomposition temperature (228 °C) than CS-extracted oil (188 °C) and SC-CO_2_-extracted oil (202 °C). Above all, SCF-extracted oil was more thermo-stable than the other oils. It also contained a lower percentage of linoleic acid (45.05%) that provided it with greater stability compared with oils extracted by CS (45.23%) and SC-CO_2_ (46.43%) [31]. Beyond this region, the DTG curve became steady and smooth where the temperature was called burn-out temperature, which stood for the temperature where sample oxidation was completed [32]. Overall, the SCF extraction method proved to be superior to the CS and SC-CO_2_ methods in terms of lower acid content, lower peroxide value, higher unsaturated fatty acid content, higher α-tocopherol content and greater thermally stability.

### 2.3. Protein Product Characterization

Table A1 shows the amino acid composition of oilseed meal (defatted by CS, SCF and SC-CO_2_ methods) protein hydrolysates. The total amount of amino acids in the CS, SCF, and SC-CO_2_-defatted meals was 496, 378 and 421 mg/g, respectively. Some amino acids, such as histidine, tyrosine, methionine, and cystine have been reported to show antioxidant properties [33]. The total amino acid contents of histidine, tyrosine, methionine, and cystine in the meals that were defatted by CS, SCF, and SC-CO_2_ accounted for approximately 10.88%, 10.57%, and 10.25%, respectively. The results presented here were similar to the results obtained in a study of the rapseed content of 11.59% [34].

The nutritive value of dietary proteins is determined by the pattern and quantity of essential amino acids (EAAs). The amino acid profiles of oilseed meals defatted by CS, SCF, and SC-CO_2_ methods were rich in EAAs (21.46-24.53%). At the same time, the EAA content of CS-defatted meal had a higher concentration than SCF and SC-CO_2_-defatted meals. Higher EAA content could provide more building blocks for protein synthesis as well as increase the synthetic rate of protein [35].

Both the nitrogen solubility index and protein dispersibility index were used to measure the solubility of the protein in water. The nitrogen solubility index (NSI) and protein dispersibility index (PDI) results are shown in Table A1. The NSI value of SCF-defatted meal (49.64%) was significantly higher (*p* < 0.05) than the values for CS (35.57%) and SC-CO_2_ (35.78%)-defatted meals. The PDI value of SCF-defatted meals (50.80%) was significantly higher (*p* < 0.05) than the values of CS (37.23%) and SC-CO_2_ (38.28%)-defatted meals. The results showed that extraction temperature had a significant effect on the solubility of oilseed meal proteins. Differences in protein solubility among the meals could be attributed to different levels of protein denaturation caused by the effect of the treatment temperature used for oil removal. Once denatured, the protein was unfolded, exposed its internal hydrophobic groups, and the hydrophobic residues exposure reduced protein solubility [36]. The higher protein content of SCF-defatted meal indicated its higher biological value for application in food, industry, and pharmaceuticals than the protein content from the CS and SC-CO_2_ methods.

### 2.4. Comparison with CS and SC-CO_2_

The oil yield (27.78%) was attained in the case of SCF extraction with *n*-butane solvent under the following conditions of extraction temperature at 56 °C and S/S ratio of 13 mL/g with a short extraction time (40 min). However, a high pressure (35 MPa) produced by SC-CO_2_ could limit the application because of the high cost at production scale, and the 17.34% oil yield indicated the low processing capacity. Though CO_2_ is the most utilized solvent, *n*-butane can be a good choice because its critical pressure is relatively lower than that of CO_2_, leading to a better interaction with the non-polar substances [37]. In addition, *n*-butane is easily accepted, cheaper and has a higher dissolving capacity compared to CO_2_, thus decreasing the *n*-butane consumption, increasing efficiency and shortening extraction time [23,28]. The *n*-hexane is the most widely utilized in industry due to its cost-effectiveness and simplicity. Despite the fact that the hexane extraction gives a higher yield (28.03%), the solvent residue left in the oil is considered to be toxic to humans and animals. Moreover, a long period of extraction (6 h) and a relatively high temperature (80 °C) during the hexane extraction process resulted in undesirable effects on the quality of the extracted oil and the defatted meal. In addition, hexane extraction is less inclined to be selected due to difficulties in the management of hexane because of its flammability and the need for an evaporation step for oil separation [38]. Solvent evaporation is not generally required in the *n*-butane extraction, without considering the solvent residues in the final oil. It was a highlight that the SCF method provided maximum preservation of the quality of the meal (high soluble protein content) and achieved the maximum utilization of the oilseed resource. All these characteristics of SCF make it very popular in the functional food, medicine and health product fields. Taking the yield and economical factors into consideration, the SCF extraction method was acceptable and used as an environmentally friendly procedure for oil extraction from Chinese quince seeds. 

## 3. Materials and Methods 

### 3.1. Material

Chinese quince seeds were collected from healthy and ripe fruits harvested in Tongbai County (latitude: 32°30′ N; longitude: 113°30′ E), Henan Province, China. The seeds used in the experiments were ground into powder, and the particles retained on the 20 mesh sieve were used in the experiments. Seed moisture, ash, protein, crude fibre, and oil content of the seeds were determined according to the American Oil Chemists’ Society (AOCS) methods. The results showed that CQS moisture, ash, protein, crude fibre, and oil contents were 5.12% ± 0.02%, 4.03% ± 0.01%, 21.76% ± 0.03%, 10.14% ± 0.01%, and 33.38% ± 0.01%, respectively. 

### 3.2. Oil Extraction Process

Subcritical fluid extraction: SCF extraction was performed using an apparatus (AY Mantianxue Food Manufacturing Co., Ltd, Anyang, China). A G445-5/6-13 pump (Beijing Huizhi Mechanical and Electrical Equipment Co., Ltd, Beijing, China) with digital readout of flow rate was used to impel the *n*-butane through the system. The extraction capacity was 5 L, and the maximum flow rate of the *n*-butane fluid was 80 L/h. For each SCF extraction, 100 g samples of pretreated seeds were used. 

Supercritical carbon dioxide extraction: SC-CO_2_ extraction was performed with an SFT 110 extractor (Supercritical Fluids, Inc., Newark, DE, USA). In all experiments, 300 g samples were used. The pressure was monitored and maintained at a constant 35 MPa, and 60 min was required for a static extraction. Then, the stop-value was opened, and the flow rate of CO_2_ was controlled at 9.6 L/min with heat for 20 min. The extractor was maintained at a constant temperature of 60 °C, and the seed oil was recovered. 

Classical Soxhlet extraction: 50 g of pre-processed seeds was loaded into a Soxhlet extractor and extracted with *n*-hexane at 80 °C for 6 h. Then, the *n*-hexane was evaporated to dryness in a rotary vacuum evaporator at 50 °C, and the seed oil was recovered.

RSM design and statistical analysis: the experimental conditions for the SCF extraction of oil from Chinese quince seeds were optimized by the BBD of RSM. For single-factor experimentation, three parameters, *X_1_* (temperature), *X_2_* (time), *X_3_* (solvent/solid ratio) were selected for the extraction experiments at three levels of variation (Table 1). The RSM data were analysed by Design-Expert software (version 8.0.6.1, Stat-Ease, Inc., Minneapolis, MN, USA).

### 3.3. Oil Characterization

Total and 2-position fatty acid composition: the samples were derivatized to fatty acid methyl esters and analysed by gas chromatography. The methylation of the fatty acids was performed according to the AOCS official method Ce 2-66. Then, 1, 3-specific pancreatic lipase was employed for analysis of the composition of the Sn-2 fatty acids according to the AOCS methods. All samples were run in triplicate [39]. 

Physico-chemical determinations: official methods of the AOCS were used for the determinations of colour (Cc13e-92) [40], density (Cc 10a-25), refraction (Cc 7-25), acid (Cd 3d-63), iodine (Cd 1-25), peroxide (Cd 8-53), unsaponification matter (Ca 6a-40), and AOCS saponification (Cd 3c-91) [41,42]. The oxidative stabilities of the oil samples were determined with the Rancimat 743 (Metrohm AG, Switzerland) following the AOCS official method Cd 12b-92 [43]. The oil samples (3.5 ± 0.1 g) were heated at 120 °C with a continuous airflow of 20 L/h passing through the sample. 

Tocopherol composition: tocopherol was analysed by a high performance liquid chromatography system (Waters e2695, Milford, MA, USA). The system was operated at a flow rate of 0.8 mL/min. Separations were carried out at 40 °C with the fluorescence detector excitation and emission wavelengths set at 298 nm and 325 nm, respectively. Calibration curves for α-, β-, γ-, δ-tocopherols were prepared at concentrations of 1–100 μg/mL with their respective standards. The analyses were carried out in duplicate, and the results were presented as the mean ± standard deviation [12]. 

Thermal stability: TG and DTG analyses were carried out using a Setaram-Labys TG/DSC thermogravimetric analyzer. Approximately 6.5 mg of the sample was heated from room temperature to 500 °C at a rate of 5 °C/min an air atmosphere. 

### 3.4. Protein Characterization

Amino acid profile analysis: under a nitrogen atmosphere, the defatted meals (50 mg) were hydrolysed with 10 mL of hydrochloric acid (6 moL /L) at 110 °C for 24 h. The filtered hydrolysate was dried in vacuum desiccators at 45 °C and redissolved in citrate buffer (pH 2.2). The solutions were injected into an automatic amino acid analyser (S-433 D, Sykam Co., Fürstenfeldbruck, Germany). Quantification and identification of amino acids were obtained from comparison of the rentention times of peaks with the retention time standards [34]. 

NSI and PDI of the meal: the NSI was determined according to the AOCS method Ba 11-65 [44]. The sample (5 g) was dissolved in distilled water (200 mL), and mixed at 120 rpm for 2 h. The mixtures were placed in an HZQ-B automatic shaking incubator (Jingda Instrument Manufacturing Co., Ltd., Jintan, China) at 30 °C. After this process, the supernatant was centrifuged (LD5-10, Jingli Co., Beijing, China) at 1500 rpm for 10 min, and the NSI of the filtrate was separated and determined by the official method. The NSI was calculated as:

(2) $$\mathrm{NSI}\;(\%)\;=\frac{\mathrm{Nitrogen}\;\mathrm{Content}\;\mathrm{in}\;\mathrm{Supernatant}}{\mathrm{Nitrogen}\;\mathrm{Content}\;\mathrm{in}\;\mathrm{Sample}}\times 100\%$$

The PDI was determined according to the AOCS method Ba 10b-09 [44]. Sample (10 g) was dissolved in distilled water (250 mL) and mixed by an Ultra-turrax homogenizer (FM200, Fluko, Shanghai Co. Ltd, Shanghai, China) for 10 min at 8,500 rpm. The supernatant was centrifuged (LD5-10, Jingli Co., Beijing, China) at 2700 rpm for 10 min, and the PDI of the filtrate was separated and determined by the official method. The PDI was calculated as:

(3) $$\mathrm{PDI}\;(\%)\;=\frac{\mathrm{Protein}\;\mathrm{Content}\;\mathrm{in}\;\mathrm{Supernatant}}{\mathrm{Protein}\;\mathrm{Content}\;\mathrm{in}\;\mathrm{Sample}}\times 100\%$$

## 4. Conclusions

RSM was successfully applied for optimization of CQS oil yield parameters by SCF, and the maximum CQS oil yield was achieved at 56 °C, 40 min, and 13 mL/g. The second order polynomial equation developed in this study showed a high correlation between observed and predicated oil recovery values. SCF-extracted oil produced good results in terms of physicochemical properties (lower acidity and peroxide value, higher oleic, linoleic acid and α-tocopherol content), and stability, and this process was more efficient and environmentally friendly than the processes using CS and SC-CO2 methods. Thus, the SCF method was very applicable to industrial products regarding chemical compositions. The defatted CQS meal from the SCF method could be more effectively used for making protein concentrates and could be more suitably used in many foods such as bakery products, cereal products, dairy products, and comminuted processed meats compared with defatted CQS meal from the CS and SC-CO2 methods.

## Figures and Tables

**Table 1:** Box–Behnken design of three variables with the Chinese quince seed oil yields ^1^.

Run NO. ^2^	Parameters and Levels			Oil Yield (%)	
	*X_1_*	*X_2_*	*X_3_*	Experimental	Predicted
1	45	30	10	16.11	16.17
2	65	30	10	21.00	21.09
3	45	50	10	19.25	19.16
4	65	50	10	18.25	18.19
5	45	40	5	12.98	12.90
6	65	40	5	13.42	13.31
7	45	40	15	19.07	19.18
8	65	40	15	22.65	22.73
9	55	30	5	16.99	17.01
10	55	50	5	15.95	16.12
11	55	30	15	24.10	23.93
12	55	50	15	24.93	24.91
13	55	40	10	26.25	26.71
14	55	40	10	27.44	26.71
15	55	40	10	26.32	26.71
16	55	40	10	26.95	26.71
17	55	40	10	26.60	26.71

^1^ *X_1_* = temperature (°C), *X_2_* = time (min), *X_3_* = solvent/solid ratio (mL/g); ^2^ Experiments were conducted in a standard order.

**Table 2:** Analysis of variance for the Chinese quince seed oil yields as the desired response.

Source ^a^	Sum of squares	DF	Mean	*p*-value	Model fit
Model	392.23	9	43.59	< 0.0001 ^b^	*R^2^* = 0.9972
*X_1_*	125.68	1	125.68	< 0.0001 ^b^	Adj *R^2^* = 0.9937
*X_2_*	3.07	1	3.07	0.0030 ^c^	CV = 1.87%
*X_3_*	66.60	1	66.60	< 0.0001 ^b^	N/A
*X_1_ X_2_*	8.67	1	8.67	0.0001 ^b^	N/A
*X_1_ X_3_*	2.46	1	2.46	0.0053 ^c^	N/A
*X_2_ X_3_*	0.87	1	0.87	0.0494 ^c^	N/A
*X_1_^2^*	139.74	1	139.74	< 0.0001 ^b^	N/A
*X_2_^2^*	22.24	1	22.24	< 0.0001 ^b^	N/A
*X_3_^2^*	64.73	1	64.73	< 0.0001 ^b^	N/A
Residual	1.09	7	0.16	N/A	N/A
Lack of fit	0.12	3	0.040	0.9132	N/A
Pure error	0.97	4	0.24	N/A	N/A
Total	393.41	16	N/A	N/A	N/A

DF = degrees of freedom; N/A = not applicable. ^a^ *X_1_* = temperature (°C), *X_2_* = time (min), *X_3_* = solvent/solid ratio (mL/g), *R^2^* = coefficient of determination, CV: coefficient of variation; ^b^ Statistically significant at *p* < 0.001; ^c^ Statistically significant at *p* < 0.05.

**Table 3:** Total and sn-2 main fatty acid composition of Chinese quince seed oils extracted by various methods ^1^.

Fatty acid	sn-1,2,3 (%)			sn-2 Position (%)		
	CS	SCF	SC-CO_2_	CS	SCF	SC-CO_2_
C14:0	0.07 ± 0.00	0.05 ± 0.00	0.06 ± 0.01	0.11 ± 0.02	0.06 ± 0.00	0.10 ± 0.01
C16:0	9.35 ± 0.27	8.96 ± 0.38	9.86 ± 0.30	0.96 ± 0.16	3.00 ± 0.72	1.62 ± 0.31
C18:0	2.80 ± 0.06	2.79 ± 0.05	2.43 ± 0.01	0.52 ± 0.18	5.83 ± 1.64	1.27 ± 0.53
C18:1	40.35 ± 0.17	40.94 ± 0.16	38.38 ± 0.09	36.62 ± 0.24	31.90 ± 0.86	32.32 ± 0.11
C18:2	45.23 ± 0.07	45.05 ± 0.05	46.43 ± 0.10	61.37 ± 0.27	58.80 ± 1.53	63.57 ± 0.72
C18:3	0.31 ± 0.01	0.27 ± 0.01	1.02 ± 0.03	0.42 ± 0.02	0.27 ± 0.00	1.12 ± 0.00
C20:0	1.17 ± 0.02	1.20 ± 0.08	1.05 ± 0.10	ND	0.14 ± 0.03	ND
C20:1	0.48 ± 0.01	0.49 ± 0.03	0.58 ± 0.01	ND	ND	ND
C22:0	0.23 ± 0.01	0.25 ± 0.03	0.19 ± 0.00	ND	ND	ND

^1^ Values are means ± s.d. of three replicate analyses. CS = classical Soxhlet, SCF = subcritical fluid, SC-CO_2_ = supercritical CO_2_, ND = not detected.

**Table 4:** Characteristics of Chinese quince seed oil extracted by various methods ^1^.

Extraction method	CS	SCF	SC-CO_2_
Colour (Lovibond units)	99.40 ± 1.15	95.50 ± 0.17	82.00 ± 1.83
Density (g/mL)	0.919 ± 0.02	0.917 ± 0.02	0.920 ± 0.03
Refractive index	1.472 ± 0.002	1.474 ± 0.012	1.473 ± 0.001
Acid value (mg KOH/g)	6.87 ± 0.12	3.97 ± 0.04	4.75 ± 0.05
Iodine value (g/100 g)	107.61 ± 1.19	108.91 ± 1.20	110.27 ± 5.13
Peroxide value (meq O_2_/kg)	0.03 ± 0.00	0.02 ± 0.00	0.04 ± 0.00
Unsaponifiable matter (%)	0.91 ± 0.001	1.35 ± 0.006	1.61 ± 0.002
Saponification value (mg KOH/g)	190.72 ± 0.79	191.25 ± 0.88	193.76 ± 0.91
Tocopherols (mg/kg)	620.1 ± 0.21	851.3 ± 0.23	578.4± 0.28
α-Tocopherol	619.9± 0.31	847.6± 0.32	576.0± 0.47
β-Tocopherol	0.2± 0.28	1.2± 0.08	1.1± 0.72
γ-Tocopherol	ND	2.5± 0.05	1.3± 0.24
δ-Tocopherol	ND	ND	ND
Induction time (h)	6.77 ± 0.04	6.85 ± 0.07	0.33 ± 0.00

^1^ Values are means ± s.d. of three replicate analyses. ND = not detected.

## Appendix

**Table A1:** Main amino acid composition (acid treated) and functional characteristics of oilseed meals defatted by various methods.

Parameter	CS	SCF	SC-CO_2_
Amino acid (%, dry weight basis)			
Asparaginic acid	11.74	11.37	11.35
Threonine	1.50	1.57	3.46
Serine	2.60	2.56	4.31
Glutamic acid	28.88	28.76	26.58
Glycine	6.79	6.79	6.88
Alanine	1.41	3.86	4.06
Cystine	4.04	3.74	3.82
Valine	3.20	2.60	2.42
Methionine	0.49	0.43	0.45
Isoleucine	2.24	2.25	2.28
Leucine	8.97	7.05	7.07
Tyrosine	3.15	3.46	3.27
Phenylalanine	5.01	4.92	3.52
Histidine	3.20	2.93	2.72
Lysine	3.12	2.64	2.48
Arginine	7.22	8.63	8.56
Proline	6.43	6.44	6.77
Nitrogen solubility index (NSI, %) ^1^	35.57	49.64	35.78
Protein dispersibility index (PDI, %) ^1^	37.23	50.80	38.28

^1^ Values are means ± s.d. of three replicate analyses.
